# Absence of autoantibodies against correctly folded recombinant fibrillin-1 protein in systemic sclerosis patients

**DOI:** 10.1186/ar1813

**Published:** 2005-09-06

**Authors:** Jürgen Brinckmann, Nico Hunzelmann, Ehab El-Hallous, Thomas Krieg, Lynn Y Sakai, Sven Krengel, Dieter P Reinhardt

**Affiliations:** 1 Department of Dermatology, University of Lübeck, Lübeck, Germany; 2 Department of Dermatology, University of Cologne, Cologne, Germany; 3 Department of Medical Molecular Biology, University of Lübeck, Lübeck, Germany; 4 Department of Biochemistry and Molecular Biology and Shriners Hospital for Children, Oregon Health and Science University, Portland, OR, USA; 5 Department of Anatomy and Cell Biology and Faculty of Dentistry, McGill University, Montreal, Canada

## Abstract

Autoantibodies against short recombinant fragments of fibrillin-1 produced in bacterial expression systems have been found in tight-skin mouse, systemic sclerosis, mixed connective tissue disease, and primary pulmonary hypertension syndrome. In patients with scleroderma, the frequency of anti-fibrillin-1 antibodies was 42% in Caucasians. Until now it has been unclear whether this immune response has a primary function in disease pathogenesis or is a secondary phenomenon. In the present study we analyzed the frequency of autoantibodies against two overlapping recombinant polypeptides spanning the N-terminal and C-terminal halves of human fibrillin-1, which were produced in human embryonic kidney (HEK-293) cells. Correct three-dimensional structures of the recombinant fibrillin-1 polypeptides were shown by electron microscopy and immunoreactivity with antibodies. Screening of fibrillin-1 antibodies was performed in 41 sera from systemic sclerosis patients and in 44 healthy controls with a Caucasian background. Microtiter plates were coated with the recombinant polypeptides of fibrillin-1 and incubated with 1:100 diluted sera. Positive binding was defined as being more than 2 SD above the mean of the control group. ELISAs showed that none of the sera of patients with systemic sclerosis contained autoantibodies against the N-terminal or C-terminal recombinant fibrillin-1 polypeptide. The data show the absence of autoantibodies against recombinant fibrillin-1 protein in Caucasian systemic sclerosis patients. Because the correct three-dimensional folding of the recombinant proteins has been substantiated by several independent methods, we conclude that autoantibodies against correctly folded fibrillin are not a primary phenomenon in the pathogenesis of systemic sclerosis.

## Introduction

Systemic sclerosis (SSc) is a connective tissue disease characterized by an excess deposition of collagen in skin and/or internal organs leading to malfunction and organ failure. The extent and progression of the fibrotic process presumably caused by the imbalance between extracellular matrix synthesis and degradation largely determines the prognosis of the disease. One hallmark of the disease is the presence of circulating autoantibodies against non-organ-specific nuclear and nucleolar antigens, which can be detected in at least 95% of patients. They include anti-centromere, anti-topoisomerase I and anti-RNA polymerase antibodies and are associated with distinct disease subtypes [1].

Heterozygous tight-skin mice (Tsk/+) are characterized by a phenotype of skin thickening and visceral fibrosis due to an increased deposition of extracellular matrix proteins in skin and organs. Furthermore, Tsk/+ mice develop lung emphysema and cardiac hypertrophy and have therefore been adopted as a potential genetic model of human SSc, cardiac hypertrophy and hereditary emphysema [2]. In a similar manner to human SSc, Tsk/+ mice produce autoantibodies against SSc-specific antigens such as topoisomerase I and RNA polymerase [3].

A duplication in the mouse fibrillin-1 gene was described for the Tsk/+ mouse, which is associated with premature death *in utero *for homozygous Tsk/Tsk animals [4]. Fibrillin-1 is one of the major structural components of microfibrils, which are extracellular supramolecular aggregates found in many elastic and non-elastic tissues (reviewed in [5]). Microfibrils are thought to be important in the assembly and organization of the elastic fibers by mediating tropoelastin deposition [6]. Fibrillin-1 and other members of the fibrillin family are repetitively aligned within microfibrils and constitute their structural backbone [7,8]. Murai and colleagues found that Tsk/+ mice spontaneously produce autoantibodies against a small recombinant protein spanning the proline-rich region of human fibrillin-1 [9]. This recombinant fragment comprises about 2% of the total fibrillin-1 molecule. Recently, the presence of autoantibodies against the same recombinant fibrillin-1 fragment has also been shown for sera from patients with SSc, localized scleroderma, mixed connective tissue disease and primary pulmonary hypertension syndrome [10-12]. Frequencies of autoantibodies showed remarkable differences between the ethnic groups studied. Choctaw American Indians and Japanese patients with SSc exhibited the highest frequency, with 81% and 78% respectively, whereas Caucasians with SSc were positive to a smaller extent with 34% [10].

In the present study we analyzed the autoantibody titer in Caucasian SSc patients against two overlapping recombinant fragments spanning the entire human fibrillin-1. One fragment constitutes the amino-terminal half of fibrillin-1 (amino acid residues 19 to 1,527) and the other fragment its carboxy-terminal half (residues 1,487 to 2,725). Before the analysis of antibody titers by ELISA, the proper folding of both recombinant proteins was shown by electron microscopy after rotary shadowing and binding of monoclonal and polyclonal antibodies by dot-blotting with or without previous reduction of the recombinant proteins.

## Materials and methods

### Patients and tissue specimens

Sera from Caucasian patients with SSc (*n *= 41; 29 female, 12 male; mean age 58.2 ± 14.3 years) and from healthy Caucasian controls (*n *= 44; 31 female, 13 male; mean age 46.9 ± 19.8 years) were studied. Patients with SSc were diagnosed in accordance with the American College of Rheumatology preliminary criteria for the classification of SSc [13]. Limited systemic sclerosis was present in 25 patients, and diffuse systemic sclerosis in 16. The range of disease duration was between 6 months and 27 years. The antibody profile showed positive titers of anti-nuclear antibodies for all patients. Of these, 16 had SCL-70, 13 anti-centromere, 1 RNA polymerase and 11 undifferentiated antibodies. Antibody testing consisted of the determination of the fluorescence pattern and titer on HEP2 cells (Viramed, Germany) as well as subsequent testing by a commercial ELISA for U1-RNP, Sm, Ro-SSA, La-SSB, Scl-70 and centromere reactivity (Orgentec, Germany). All samples were obtained after obtaining written consent from the donors under protocols approved by the local ethical committee.

### Expression and production of recombinant fibrillin-1 polypeptides

The expression plasmids to express the N-terminal half (pDNSP-rF16) and the C-terminal half (pcDNA-rF6H) of human fibrillin-1 have previously been described in detail [14]. On the basis of SDS-PAGE and electron microscopy after rotary shadowing (see below), the purity of the recombinant fragments was more than 90%. Stable clones with these expression plasmids were obtained with human embryonic kidney (HEK-293) cells as described in detail [15]. The expression of pDNSP-rF16 in eukaryotic cells produces a secreted polypeptide (rF16) with the sequence Ala-Pro-Leu-Ala-Ser^19^-Val^1,527^-(His)_6_. The expression of pcDNA-rF6H in eukaryotic cells produces a secreted polypeptide (rF6H) with the sequence Ala-Pro-Leu-Ala-Asp^1,487^-Lys^2,725^-(His)_6_. Production and purification of rF16 and rF6H were performed as in the procedures described elsewhere [16].

### Electron microscopy after rotary shadowing

The purified proteins were adjusted to a concentration of 0.25 mg/ml and dialyzed against 100 mM NH_4_HCO_3_. The samples were diluted with 0.05% (v/v) acetic acid to a final protein concentration of 60 μg/ml and mixed with glycerol to a final concentration of 50% (v/v) glycerol. Then 80 μl of the samples was sprayed onto freshly cleaved mica from a distance of 25 cm and dried under high vacuum (about 9 nbar) for about 2 to 3 hours in an Edwards Auto 306 vacuum coater. Rotary shadowing was performed by platinum evaporation for 15 s at 50 mA and 2.5 kV at an angle of 5° and a distance of 12 cm. The samples were rotated at 120 r.p.m., followed by coating with coal for stabilization for 2 s at 100 mA and 2.5 kV at an angle of 90°. The replicas were floated onto a very clean surface of distilled water and then supported with 400-mesh copper grids. Replicas were examined at 100 kV in a transmission electron microscope (Zeiss TEM 109).

### Cell culture

Human dermal fibroblasts were derived from explant cultures of dissected tissues obtained from surgical samples after informed consent had been obtained. The cells were cultured in DMEM supplemented with 10% fetal bovine serum and penicillin/streptomycin (Invitrogen). Cells (10^6^) were plated in a 60 mm dish and grown for 72 hours. The cell layers were washed with phosphate-buffered saline and then incubated for 24 hours in 3 ml of DMEM without serum. The conditioned medium was harvested and treated with 1 mM phenylmethylsulfonyl fluoride.

### Dot-blot assay

Either 2 μg of purified recombinant proteins rF16 and rF6H or 1 ml of conditioned medium were transferred to nitrocellulose membranes using a dot-blot apparatus (Bio-Rad) with or without previous reduction of the proteins with 0.05 M dithiothreitol. After staining with Ponceau S, non-specific binding sites on the nitrocellulose membrane were blocked for 1 hour with Tris-buffered saline (TBS) containing 5% (w/v) non-fat milk. Nitrocellulose membranes were probed with a polyclonal antiserum against rF6H (diluted 1:500 [17]) and with monoclonal antibodies directed against rF6H (mAb 69, about 4 μg/ml) and rF16 (mAb 201 and mAb 26, both about 4 μg/ml [18]) followed by peroxidase-conjugated anti-rabbit or anti-mouse secondary antibody (diluted 1:800; Bio-Rad). Bound antibodies were revealed in accordance with the manufacturer's instructions by using the horseradish peroxidase developer 4-chloronaphthol (Bio-Rad).

### ELISA assay

Microtiter plates were coated with 100 μl of 10 μg/ml purified recombinant human fibrillin-1 fragments rF16 and rF6H or BSA overnight at 4°C. After being washed three times with TBS containing 0.05% Tween 20 (TBS/Tween), the plates were blocked for 1 hour with 200 μl of 5% non-fat milk powder in TBS at room temperature (20°C). After being washed with TBS, the plates were incubated for 2 hours with 100 μl of test sera diluted 1:100 with TBS containing 5% non-fat milk powder at room temperature. After being washed three times with TBS/Tween, the plates were incubated for 1.5 hours with 100 μl of the horseradish peroxidase-conjugated secondary antibody (diluted 1:800) at room temperature (goat anti-rabbit for positive control sera, and goat anti-human for human sera; Sigma, Germany). After three washings with TBS/Tween, color development was achieved with 100 μl of 1 mg/ml 5-aminosalicylic acid in 0.02 M phosphate buffer (pH 6.8) and 1.5 μl/ml H_2_O_2_. Color development was stopped after 1 hour by the addition of 100 μl of 2 M NaOH. Absorbance was measured at 492 nm with an ELISA reader (Anthos, Austria). All experiments were run in parallel triplicates; the ELISA test was performed twice. The background binding of serum antibodies to BSA-coated wells was subtracted from the binding of serum to the respective rF16-coated and rF6H-coated wells after subtraction of the respective background of rF16-coated, rF6H-coated and BSA-coated blanks. Positive binding was defined as more than 2 SD above the mean of the control sera. The coefficient of variation was 7.4% (*n *= 10).

## Results

### Ultrastructural analysis of recombinant fibrillin-1 polypeptides

**Figure 1:**
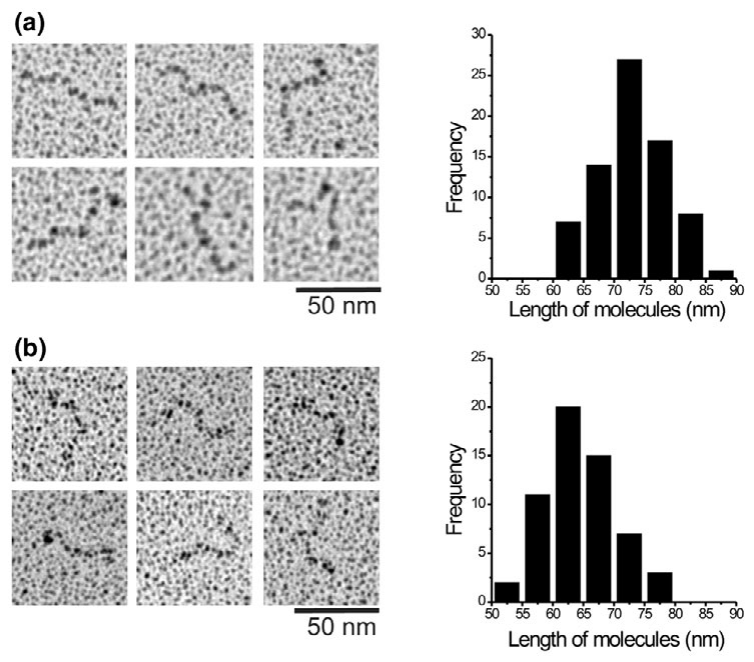
Recombinant amino-terminal (rF16)**(a)** and carboxy-terminal (rF6H)**(b)** halves of human fibrillin-1 were analyzed by electron microscopy after rotary shadowing. Representative images and histrograms of the measured lenghs of the recombinant fragments are shown. Note that both fragments showed thread-like extended molecules. The measurements are plotted as number of measurements, in 5 nm windows. The average length of rF16 was 73.1 ± 5.7 nm (mean ± SD; *n *= 75) and the average length of rF6H was 64.2 ± 5.9 nm (mean ± SD; *n *= 56).

To analyze the molecular shape of the recombinant polypeptides, they were revealed by electron microscopy after rotary shadowing (Fig. 1). These results showed thread-like extended molecules for the recombinant polypeptides rF16 and rF6H representing the N-terminal and C-terminal halves of human fibrillin-1. At the termini of rF16 and rF6H the molecules occasionally adopted a curved shape.

The analysis of molecular dimensions revealed that the length of rF16 (73.1 ± 5.7 nm, *n *= 75) and rF6H (64.2 ± 5.9 nm, *n *= 56) corresponded well to the lengths for very similar constructs described previously [16] as well to the respective parts in full-length fibrillin-1 [19]. The extended shape of the recombinant proteins is a very good indicator of correct folding, because the molecular shape is determined by numerous intramolecular disulfide bridges stabilizing this extended structure [20,21].

### Immunoreactive analysis of recombinant fibrillin-1 polypeptides

**Figure 2:**
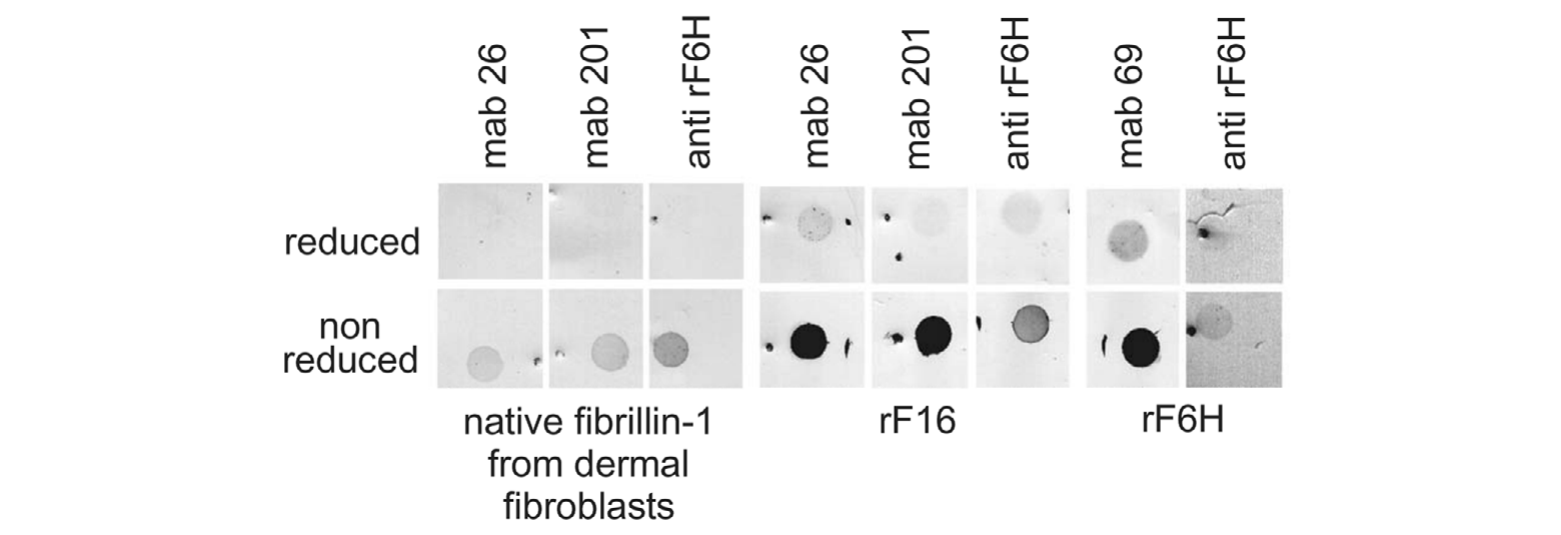
Immunoreactive analysis of fibrillin-1 antibodies against recombinant fibrillin-1 polypeptides and against native fibrillin-1. Purified recombinant amino-terminal (rF16) and carboxy-terminal (rF6H) halves of human fibrillin-1 (2 μg of each) or 1 ml of conditioned medium (containing less than about 0.2 μg of fibrillin-1) produced by human dermal fibroblasts were transferred to nitrocellulose membranes with (upper panel) or without (lower panel) previous reduction by dithiothreitol. Nitrocellulose membranes were probed with a polyclonal antibody against rF6H (anti-rF6H) or with monoclonal antibodies (mAbs) 26, 201, and 69 directed against rF16 and rF6H. The dot-blots show that the binding of all antibodies depends markedly on the presence of disulfide bonds, which are crucial for the proper folding of epitopes in both native fibrillin-1 and the recombinant fragments.

To analyze the immunoreactive properties of native fibrillin-1 synthesized by human dermal fibroblasts and the recombinant polypeptides rF16 and rF6H, dot-blotting under reducing and non-reducing conditions was performed (Fig. 2) Native fibrillin-1 reacts with monoclonal antibodies mAb 26 or mAb 201 or with polyclonal antibody anti-rF6H only under non-reducing conditions (not under reducing conditions). These data show that the antibodies primarily recognize epitopes in the correctly folded fibrillin-1 molecule but not in the denatured fibrillin-1 molecule. When the recombinant fibrillin-1 polypeptides rF16 and rF6H were tested in this assay, they showed much more reactivity in the non-reduced conformation than in the reduced conformation, showing that the corresponding epitopes are present in the same correct conformation as in native fibrillin-1. These data substantiate that the recombinant polypeptides are correctly folded.

### ELISA analysis of sera from patients and controls by using rF16 and rF6H

**Figure 3:**
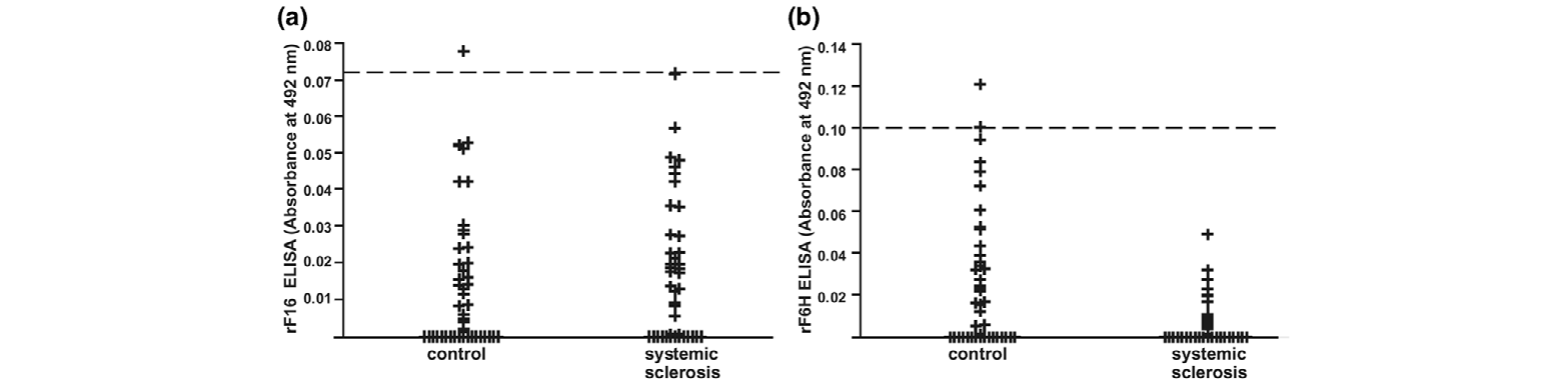
Analysis of immunoreactivity of sera from systemic sclerosis patients and healthy controls of Caucasian origin. ELISA assays with the recombinant amino-terminal (rF16 **(a)**) and carboxy-terminal (rF6H **(b)**) halves of human fibrillin-1 are shown. Microtiter plates were coated with purified rF16 and rF6H or bovine serum albumin. The plates were incubated with test sera diluted 1:100 for 2 hours at room temperature. After incubation with horseradish peroxidase-conjugated secondary antibody and color development, the absorbance was determined by an ELISA reader. Positive binding was defined as more than 2 SD above the mean (dashed line) of the control sera. If the blank value exceeded the sample value the absorbance was set to zero in the figure. None of the sera showed a positive reactivity to rF16 or rF6H.

A cutoff value was established for each ELISA as a value of 2 SD above the mean of 44 control sera. For rF16 the cutoff ELISA score was 0.072 and for rF6H it was 0.1. The analysis of 41 sera from Caucasian patients with systemic sclerosis showed that none of the sera exceeded the cutoff value for the N-terminal half of fibrillin-1. Furthermore, the ELISA score of all sera tested for the presence of antibodies against the C-terminal half of fibrillin-1 was in the normal range of the controls (Fig. 3).

## Discussion

Mutations in the gene encoding fibrillin-1 have been documented for Marfan syndrome and some related disorders in humans, and for Tsk in animals [22,4]. The Tsk mutation in the fibrillin-1 gene, a 30-kilobase gene duplication of exons 17 to 40 containing a long centrally located stretch of calcium-binding epidermal growth factor-like domains, is accompanied by premature death *in utero *in homozygous mice, whereas mice heterozygous for the duplication are viable and show the tight-skin phenotype. The mutation results in a larger protein (418 kDa, as compared with 350 kDa in normal animals) which after incorporation along with wild-type fibrillin-1 seems to render all microfibrils more susceptible to proteolysis [23]. In a similar manner to SSc in humans, Tsk mice develop autoimmunity with antibodies against topoisomerase I and RNA polymerase. Recently, autoantibodies against a small 30 kDa human recombinant fibrillin-1 polypeptide covering the proline-rich region (residues 395 to 446) have been detected in 41% of Tsk mice [9].

Autoantibodies against the same recombinant fibrillin-1 polypeptide were also found in humans affected by SSc or primary pulmonary hypertension syndrome [10,12]. Especially in SSc, the frequency of anti-fibrillin-1 antibodies and the recognized epitopes differ according to the ethnic background of patients, as shown in a subsequent study [24]. In that study, reactivity against recombinant polypeptides covering the N-terminal end (residues 15 to 193), the proline-rich region (residues 367 to 425), and a stretch of calcium-binding epidermal growth factor-like domains (residues 1,326 to 1,549) was tested. Taking the different epitopes tested in that study together, Choctaw Native Americans, Japanese patients and African Americans revealed the highest levels with 100% and 80%, respectively. In the same study, sera from Caucasian SSc patients showed the presence of anti-fibrillin-1 antibodies in 42% of patients. Whether the occurrence of these autoantibodies has a primary role in the pathogenesis of SSc or is a secondary phenomenon is open to discussion.

In our study of 41 Caucasian patients with SSc, none of the sera showed positive reactivity against the recombinant polypeptide spanning either the N-terminal half or the C-terminal half of fibrillin-1. Structural studies by rotary shadowing and evaluation of molecular lengths showed that the recombinant fibrillin-1 polypeptides used resemble native molecules. They adopt the correct dimensions and extended conformations similar to regions observed in whole molecules of native fibrillin-1 purified from cell culture medium [19]. Various monoclonal and polyclonal antibodies recognize native fibrillin-1 only in a non-reduced (correctly folded) conformation but not in the reduced (misfolded) conformation because numerous intramolecular disulfide bonds stabilize the native conformation of fibrillins [20,21]. Similar binding properties of these monoclonal and polyclonal antibodies to the recombinant polypeptides rF16 and rF6H strongly support the notion that these polypeptides are folded correctly. Our data clearly show that SSc in Caucasians is not characterized by the presence of autoantibodies against properly folded fibrillin-1. This observation indicates that the presence of autoantibodies against fibrillin-1 does not have a primary role in the pathogenesis of the disease.

The recombinant fibrillin-1 antigens used in other studies showing a positive binding of antibodies obtained from SSc patients were relatively small (59, 179 and 224 residues) and were produced in bacterial expression systems [10,24]. No structural or functional characterization for these recombinant polypeptides is available to determine whether they adopt native or misfolded conformations. It is possible that the anti-fibrillin-1 autoantibodies detected with such recombinant polypeptides recognize cryptic or misfolded antigenic epitopes for example, which may become available after proteolytic fragmentation of fibrillin-1 in SSc or may be antibodies against cross-reacting antigens. In this light, one can speculate that these autoantibodies are a secondary phenomenon in SSc. This interpretation is further substantiated by a metabolic analysis of fibrillin-1 synthesized by SSc fibroblasts in cell culture, which revealed decreased amounts of abnormal microfibrils [25]. Furthermore, in the same study *in vitro*, data indicated that the amount of fibrillin-1 in the extracellular matrix produced by SSc cells diminished faster than in the matrix of control cells, arguing for a higher susceptibility to proteolytic degradation.

## Conclusion

Our data clearly show that sera from 41 Caucasian SSc patients contained no autoantibodies against properly folded recombinant human fibrillin-1. These data therefore provide evidence that autoimmunity against fibrillin-1 is a secondary phenomenon in the pathogenesis of SSc in Caucasians.

## Abbreviations

BSA = bovine serum albumin; DMEM = Dulbecco's modified Eagle's medium; ELISA = enzyme-linked immunosorbent assay; kDa = kilodaltons; mAb = monoclonal antibody; SSc = systemic sclerosis; TBS = Tris-buffered saline; Tsk = tight-skin mouse.

## Competing interests

The author(s) declare that they have no competing interests.

## Authors' contributions

Expression and production of recombinant polypeptides were performed by EE and DPR. Electron microscopy was performed by EE and DPR. ELISA analysis was performed JB, DPR, SK and NH. Immunoreactive analysis was performed by JB, LYS and DPR. Study design and coordination were performed by JB, NH and DPR. Editing of the manuscript was performed by JB, NH, DPR, TK and LYS. All authors read and approved the final manuscript.
